# HDAC Inhibitor-Mediated Epigenetic Regulation of Glaucoma-Associated TGFβ2 in the Trabecular Meshwork

**DOI:** 10.1167/iovs16-19446

**Published:** 2016-07-12

**Authors:** Jaclyn Y. Bermudez, Hannah C. Webber, Gaurang C. Patel, Xiangyang Liu, Yi-Qiang Cheng, Abbot F. Clark, Weiming Mao

**Affiliations:** 1 North Texas Eye Research Institute University of North Texas Health Science Center, Fort Worth, Texas, United States; 2 UNT System College of Pharmacy, University of North Texas Health Science Center, Fort Worth, Texas, United States

**Keywords:** glaucoma, TGFβ2, epigenetics, trabecular meshwork, ocular hypertension

## Abstract

**Purpose:**

Elevated intraocular pressure (IOP) in primary open-angle glaucoma (POAG) results from glaucomatous damage to the trabecular meshwork (TM). The glaucoma-associated factor TGFβ2 is increased in aqueous humor and TM of POAG patients. We hypothesize that histone acetylation has a role in dysregulated TGFβ2 expression.

**Methods:**

Protein acetylation was compared between nonglaucomatous TM (NTM) and glaucomatous TM (GTM) cells using Western immunoblotting (WB). Nonglaucomatous TM cells were treated with 10 nM thailandepsin-A (TDP-A), a potent histone deacetylase inhibitor for 4 days. Total and nuclear proteins, RNA, and nuclear protein-DNA complexes were harvested for WB, quantitative PCR (qPCR), and chromatin immunoprecipitation (ChIP) assays, respectively. Paired bovine eyes were perfused with TDP-A versus DMSO, or TDP-A versus TDP-A plus the TGFβ pathway inhibitor LY364947 for 5 to 9 days. Intraocular pressure, TM, and perfusate proteins were compared.

**Results:**

We found increased acetylated histone 3 and total protein acetylation in the GTM cells and TDP-A treated NTM cells. Chromatin immunoprecipitation assays showed that TDP-A induced histone hyperacetylation associated with the TGFβ2 promoter. This change of acetylation significantly increased TGFβ2 mRNA and protein expression in NTM cells. In perfusion-cultured bovine eyes, TDP-A increased TGFβ2 in the perfusate as well as elevated IOP. Histologic and immunofluorescent analyses showed increased extracellular matrix and cytoskeletal proteins in the TM of TDP-A treated bovine eyes. Cotreatment with the TGFβ pathway inhibitor LY364947 blocked TDP-A–induced ocular hypertension.

**Conclusions:**

Our results suggest that histone acetylation has an important role in increased expression of the glaucoma-associated factor TGFβ2. Histone hyperacetylation may be the initiator of glaucomatous damage to the TM.

Glaucoma is a group of optic neuropathies that lead to vision loss and irreversible blindness. The most prevalent type of glaucoma is primary open-angle glaucoma (POAG). Nearly 3 million Americans are affected by this disease and over 70 million individuals are affected worldwide.^1,2^ The number of glaucoma patients worldwide will increase to 76 million by the year 2020 and to over 111 million by 2040.^2^ In POAG patients, elevated intraocular pressure (IOP) is the major risk factor for the development and progression of this disease.^3^

Intraocular pressure is regulated by the production and egress of aqueous humor (AH), a fluid that nurtures avascular anterior segment ocular tissues. Higher outflow resistance at the trabecular meshwork (TM) leads to elevated IOP in individuals with POAG. The TM is a multilayered tissue at the iridocorneal angle of the eye. The TM consists of TM cells and extracellular matrix (ECM) beams. Glaucomatous damage to the TM results in higher AH outflow resistance and, therefore, causes IOP elevation. One of the most studied pathologic changes in the glaucomatous TM (GTM) is excessive deposition of ECM material, which is believed to “clog” the TM and decrease AH outflow.

Recently, several glaucoma-associated factors have been identified. One important factor is transforming growth factor-β2 (TGFβ2), an activator of the TGFβ signaling pathway. Transforming growth factor-β2 is a secreted protein that, when activated, binds to the TGFβ receptor type II. The binding activates TGFβ receptor type I, which phosphorylates receptor Smad proteins, Smad2/3. Phospho-Smad2/3 complexes with the co-Smad protein, Smad4, translocates into the nucleus, and regulates gene expression. TGFβ2 also signals using non-Smad pathways, including ERK 1/2, JNK, and p38. Many studies have shown that the expression of TGFβ2 is significantly higher in the glaucomatous AH as well as GTM tissues.^4–6^ Activation of the TGFβ pathway by elevated TGFβ2 leads to increased production of ECM, cytoskeletal changes, and altered cell adhesion in the TM.^7–10^ All these TGFβ2-induced changes in the TM also are found in the GTM,^8,11,12^ suggesting that TGFβ2 not only is POAG-associated, but also a causative factor of this disease. In mouse eyes as well as perfusion-cultured porcine and human eyes, overexpression of activated TGFβ2 or treatment with recombinant TGFβ2 elevates IOP, which leads to POAG pathology.^13–16^

Although TGFβ2 and other glaucoma-associated factors have been identified and studied for years, the regulatory mechanism that causes high ocular TGFβ2 expression is unknown. We have found that cultured GTM cells retain glaucomatous phenotypes including increased production of TGFβ2 and ECM as well as a reorganized actin cytoskeleton.^6,7,10,17^ Since cultured TM cells do not receive glaucomatous clues from the extracellular environment, it seems an internal mechanism is able to maintain these glaucomatous phenotypes. We believe epigenetic regulation is involved in increased production of TGFβ2.

Epigenetic regulatory mechanisms affect gene expression without altering the genomic DNA sequence and include DNA methylation, histone modification, and RNA interference. Epigenetics has been studied extensively in development, cancers, and age-related neurodegenerative diseases, such as Alzheimer's, Parkinson's, and dementia.^18–20^ However, the role of epigenetics in glaucoma, the leading cause of blindness worldwide, is unclear.

We previously reported that DNA methylation is not responsible for regulating another glaucoma-associated factor, sFRP1.^21^ Gonzales et al.^22^ reported that transfection with miR-29b mimics decreased TGFβ2 in human TM cells at the RNA level, but had little effect on TGFβ2 at the protein level. Therefore, we hypothesize that histone acetylation is responsible for the increased expression of TGFβ2 in the GTM. Our strategy was to use a novel histone deacetylase inhibitor (HDACi), thailandepsin-A (TDP-A) to inhibit HDAC activities and enhance histone acetylation. This natural compound inhibits multiple classes of HDACs with high efficacy at nanomolar concentrations, which theoretically will minimize off-target effects.^23^ We compared protein acetylation between primary nonglaucomatous human TM (NTM) and GTM cells, as well as studied the effect of altering histone acetylation on the expression of TGFβ2 and IOP in primary NTM cell cultures and perfusion-cultured bovine eyes, respectively. Our results suggest that histone hyperacetylation may initiate glaucomatous changes in the TM. Our study is the first to show that dysregulation of histone acetylation may be one of the initiators of IOP elevation, suggesting its important role in the pathology of POAG.

## Materials and Methods

### NTM and GTM Cell Cultures

Nonglaucomatous TM and GTM cells were characterized as described in our previous studies.^6,21,24–26^ Briefly, TM tissues were dissected carefully from donor eyes avoiding non-TM tissue contamination and placed in culture medium on cell culture plates. After a few days, TM cells migrated from TM explants onto culture plates. All TM cells were characterized with a panel of TM markers including collagen IV, laminin, α-smooth muscle actin, dexamethasone-induced myocilin expression, and formation of cross-linked actin networks. Age and sex information for these NTM and GTM cells are listed in Table 1. All HTM cells were used before senescence (less than 20 passages). Nonglaucomatous TM and GTM cells were cultured in Dulbecco's modified Eagle's low glucose medium (DMEM; Sigma-Aldrich Corp., St. Louis, MO, USA) containing 10% fetal bovine serum (FBS; Atlas Biologicals, Collins, CO, USA), 1% penicillin + streptomycin (Sigma-Aldrich Corp.), and 2 mM L-glutamine (GE Healthcare Life Sciences, Logan, UT, USA).

**Table 1:**
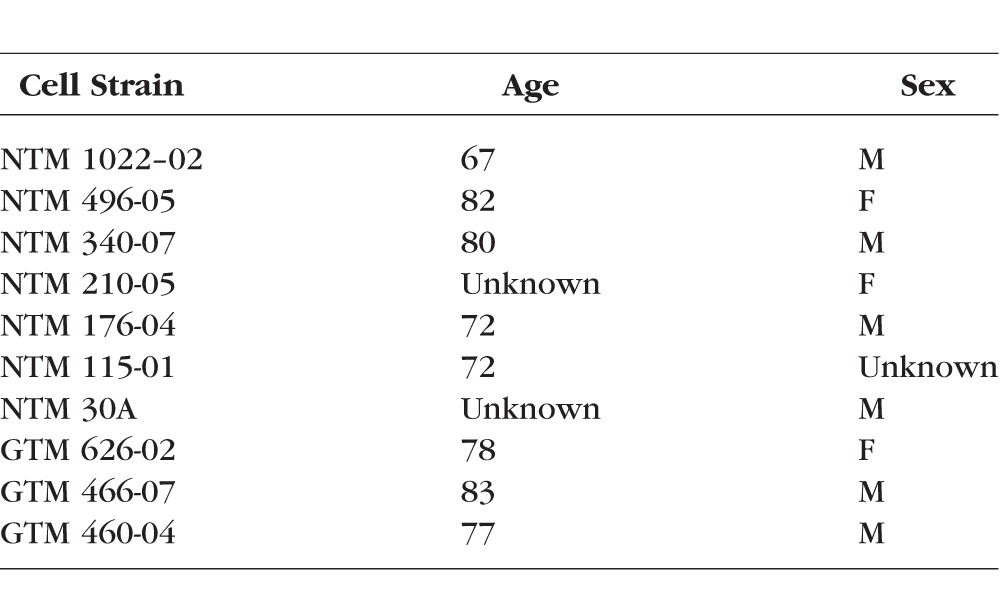
Cell Strain Information

### Western Immunoblotting (WB)

Three primary NTM and three primary GTM cell strains were cultured to confluency. Whole cell lysates and nuclear extracts were collected using the Mammalian Protein Extraction Reagent (M-PER; Thermo Fisher Scientific, Asheville, NC) and Nuclear and Cytoplasmic Extraction Reagent Kit (NE-PER; Thermo Fisher Scientific) according to the manufacturer's protocol, respectively. After protein estimation using the DC method (Bio-Rad, Hercules, CA, USA) or the EZQ method (Thermo Fisher Scientific, Waltham, MA, USA), proteins were separated using SDS-PAGE followed by transfer onto polyvinylidene difluoride (PVDF) membranes.

Whole cell lysate blots were probed with a rabbit polyclonal antibody against acetylated lysine residues (1:500; Cell Signaling Technology, Danvers, MA, USA) as well as a rabbit anti-GAPDH monoclonal antibody (1:1000; Cell Signaling Technology). Nuclear extract was used to detect acetylated histone 3 lysine 9/14 (Ac-H3-K9/K14) with a rabbit polyclonal antibody (1:500; Cell Signaling Technology), total histone 3 with a mouse monoclonal antibody (1:500; Abcam, Cambridge, MA, USA), and Lamin A/C with a mouse monoclonal antibody (4C11, 1:1000; Cell Signaling Technology).

Some NTM cells were cultured to confluency and were treated with 1% DMSO (Thermo Fisher Scientific) as a vehicle control or 10 nM TDP-A (purified at University of North Texas Health Science Center by Yi-Qiang Cheng and Xiangyang Liu^23^) for 4 days. Whole cell lysates, nuclear proteins, and conditioned medium were collected. Conditioned medium from cell cultures was concentrated with resin (StrataClean; Agilent Technologies, Santa Clara, CA, USA) at 1:100 (vol/vol). Resin was precipitated by centrifugation, and supernatant was removed. Resin was mixed with Laemmli buffer and boiled to release absorbed proteins. Whole cell lysate, nuclear extract, and enriched conditioned medium were resolved by SDS-PAGE and transferred to PVDF membranes. The blots were probed with anti-Ac-H3-K9/K14 (1:1000), total histone 3, GADPH, Lamin A/C, and/or TGFβ2 (mouse monoclonal antibody, 1:500; Abcam) antibodies.

After incubation with primary antibodies, the blots were incubated with a secondary anti-rabbit or anti-mouse antibody conjugated with HRP (Cell Signaling Technology). Signals were developed using the SuperSignal West Femto Maximum Sensitivity Substrate (Thermo Fisher Scientific) or Clarity Western ECL substrate (Bio-Rad). Images were taken using the FluroChem 8900 imager (Alpha Innotech; Biomedical Solutions, Stafford, TX, USA) or the ChemiDocTM Touch imaging system (Bio-Rad). Densitometry was performed using Image J (National Institutes of Health [NIH], Bethesda, MD, USA).

### Quantitative PCR (qPCR)

Six well-characterized NTM cell strains were cultured to confluency, and treated with 1% dimethyl sulfoxide (DMSO) or 10 nM TDP-A for 4 days. RNA was extracted using an RNA purification kit (RNeasy Mini Kit; Qiagen, Valencia, CA, USA) with DNase I treatment for 15 minutes. RNA was quantified using the NanoDrop 2000 (Thermo Fisher Scientific). RNA was reversed transcribed into cDNA using the iScript cDNA synthesis kit (Bio-Rad). Quantitative PCR was performed using the SSoAdvanced SYBR Green Supermix (Bio-Rad) in a total volume of 20 μL in a CFX96 thermocycler (Bio-Rad). The thermoprofile consisted of 40 cycles of 95°C for 10 seconds, 60°C for 30 seconds, followed by a dissociation curve. Transforming growth factor-β2 and GAPDH PCR primer (Sigma-Aldrich Corp.) sequences were: TGFβ2 forward: 5′-AGAGTGCCTGAACAACGGATT-3′,^27^ TGFβ2 reverse: 5′-CCATTCGCCTTCTGCTCTT-3′, GAPDH forward: 5′-GGTGAAGGTCGGAGTCAAC-3′,^28^ GAPDH reverse: 5′-CCATGGGTGGAATCATATTG-3′.

Data were analyzed using Wilcoxon matched-pairs signed rank test with GraphPad Prism 5 software (GraphPad, La Jolla, CA, USA).

### Chromatin Immunoprecipitation (ChIP) Assay

Three primary NTM cell strains were grown to confluency in 60-mm dishes, treated for 4 days with 1% DMSO or 10 nM TDP-A, and used for ChIP assay analysis. The SimpleChIP Enzymatic Chromatin IP Kit (Cell Signaling Technology) was used following the manufacture's protocol. Anti-acetylated histone 3 (K9/K14) antibody (Cell Signaling Technology) was used to pull down DNA-protein complexes, and rabbit IgG was used as a control. Purified DNA was quantified by qPCR. Polymerase chain reaction primer (Sigma-Aldrich Corp.) sequences for the amplification of the TGFβ2 promoter were: TGFβ2 forward: 5′-GGGAGGCTGTGACTGAGCTA-3′ and TGFβ2 reverse: 5′-GTGGGTAAGGGAGGAAGGAG-3′.

The fold of enrichment method was used for data analysis.

### Bovine Anterior Segment Perfusion Organ Culture

The experimental details for this model have been described previously.^24^ Briefly, paired bovine eyes were obtained from a local abattoir and transported to the laboratory on ice within 6 hours of sacrifice. After removal of extraocular tissues and sterilization with Betadine (Purdue Products, Stamford, CT, USA) for 2 minutes, eyes were rinsed twice with PBS. Bovine eyes were scored along the equator and the anterior segment was dissected with scissors. The vitreous, iris, uveal tract, and lens were removed carefully without disturbing the TM. The anterior segment with the cornea, sclera, and TM was mounted on a custom made Plexiglass dish. A Plexiglass O-ring was used to clamp the anterior segment at the equator to the dish using four plastic screws. This creates a watertight artificial anterior chamber. The dishes have two embedded cannulas: one allows medium infusion and the other is connected to a pressure transducer (ADInstruments, Colorado Springs, CO, USA) to measure IOP. DMEM-high glucose medium (Thermo Fisher Scientific) containing 1% glutamine, 1% penicillin + streptomycin, and 1% amphotericin B (Sigma-Aldrich Corp.) was used as the perfusion medium. A syringe pump (PHD2000; Harvard Apparatus, Holliston, MA, USA) was used to infuse medium at a constant infusion rate of 5 μL/min. Pressure transducers were connected to a data acquisition system (PowerLab; ADInstruments) consisting of a signal amplifier, a bridge amplifier, and a computer with the LabChart software (ADInstruments).

Bovine anterior segments were perfused until stable baseline IOPs were established, which usually takes approximately 24 hours. We then treated paired anterior segments for 5 to 9 days. There were two study groups: Group1 – 1% DMSO versus 10 nM TDP-A and Group2 – 10 nM TDP-A versus 10 nM TDP-A plus 5 μM inhibitor LY364947 (small molecule TGFβ Receptor I inhibitor; Tocris Biosciences, Ellisville, MO, USA).

Intraocular pressures were monitored and recorded every minute. Data for the 12 hours before treatment were averaged and used as baseline IOP. Intraocular pressure data after treatment were averaged every 24 hours for analysis. The change in IOP (ΔIOP) was defined as IOP after treatment minus baseline IOP, and the highest value after treatment was defined as maximum ΔIOP (maxΔIOP). Maximum ΔIOP was analyzed within each group using the Student's paired *t*-test since the eyes were paired. *P* values less than 0.05 were considered statistically significant.

At the end of treatment, conditioned medium (perfusate) was collected from perfusion culture dishes and spun at 500*g* for 5 minutes to remove tissue debris. Equal volumes of conditioned medium were used for WB as described previously. The primary antibody used was mouse monoclonal anti TGFβ2 antibody (1:500; Abcam).

### Histology and Immunofluorescence

Perfusion-cultured bovine anterior segments were fixed with 4% paraformaldehyde in PBS, washed three times with PBS, dehydrated with ethanol, and embedded in paraffin. Samples were sectioned at 5 μm and stained with hematoxylin and eosin (H&E) for general evaluation or Masson's trichrome stain to study total collagen.

For immunofluorescent staining, tissue sections were deparaffinized, rehydrated, and subjected to antigen retrieval using Tris-EDTA buffer (pH 9.0) in an antigen retriever (Electron Microscopy Sciences, Hatfield, PA, USA) following manufacturer's protocol. After antigen retrieval, sections were washed with PBS, incubated with 0.5% Triton X-100 in PBS, and blocked with SuperBlock (PBS) Blocking Buffer (Thermo Fisher Scientific). Tissue sections were incubated with the anti-fibronectin rabbit polyclonal antibody (1:50; Abcam) or anti-α-smooth muscle actin rabbit polyclonal antibody (α-SMA, 1:50; Abcam) overnight at 4°C, washed, incubated with the goat-anti-rabbit secondary antibody conjugated with Alexa-488 (1:200, Thermo Fisher Scientific), washed, and mounted with ProLong Gold Antifade Mountant containing 4′,6-diamidino-2-phenylendole (DAPI; Thermo Fisher Scientific).

Images were taken using the Nikon Ti inverted microscope equipped with bright field imaging setting or long-pass filters and the Nuance Multispectral Imaging System (PerkinElmer, Waltham, MA, USA). The Nuance system uses a series of images taken at different wavelengths to create individual spectral libraries for each fluorochrome or autofluorescence (background). Based on the difference in their spectral library profiles, fluorochromes are separated from each other, and autofluorescence can be subtracted.

## Results

### Protein Hyperacetylation in GTM Cells

**Figure 1:**
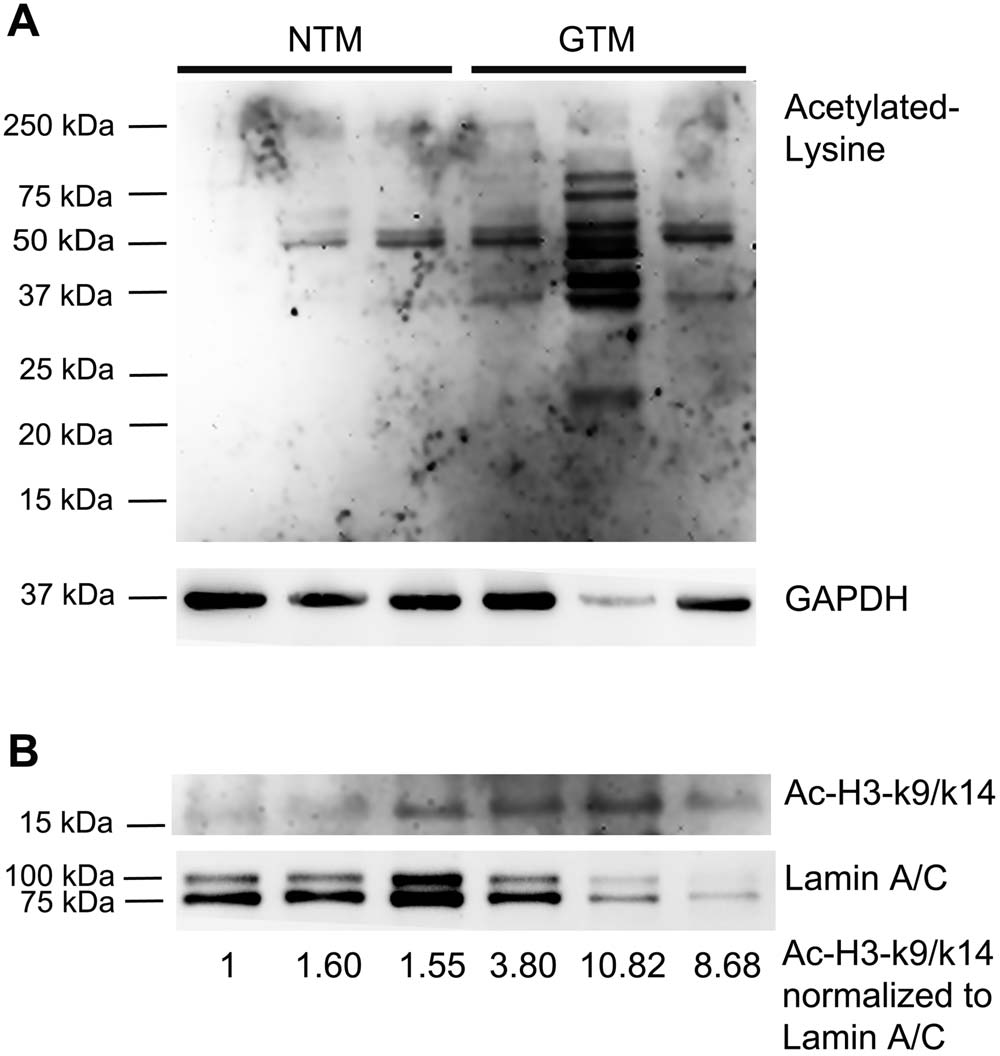
Increased protein acetylation in GTM cells. Western immunoblots of untreated NTM (1022–02, 340-07, 176-04) and GTM (626-02, 466-07, 460-04) cells. (**A**) Whole cell lysate was probed with an antibody against proteins with acetylated lysine residues, and GAPDH was used as a loading control. (**B**) Nuclear proteins were probed with antibodies against Ac-H3-K9/K14, and Lamin A/C was used as a nuclear protein loading control. The ratio of Ac-H3-K9/14 to Lamin A/C was calculated using densitometry and listed at the *bottom* of (**B**).

We first compared whether there is a difference in acetylated histone 3 and total protein acetylation between NTM and GTM cells. Three NTM and three GTM cell strains were cultured to confluency, and whole cell lysate and nuclear protein were harvested for WB. We found that there was hyperacetylation in total proteins (Fig. 1A) including the lysine residues 9 and/or 14 of histone 3 (Fig. 1B) in GTM cells.

### TDP-A Increased Protein Acetylation in NTM Cell Cultures

**Figure 2:**
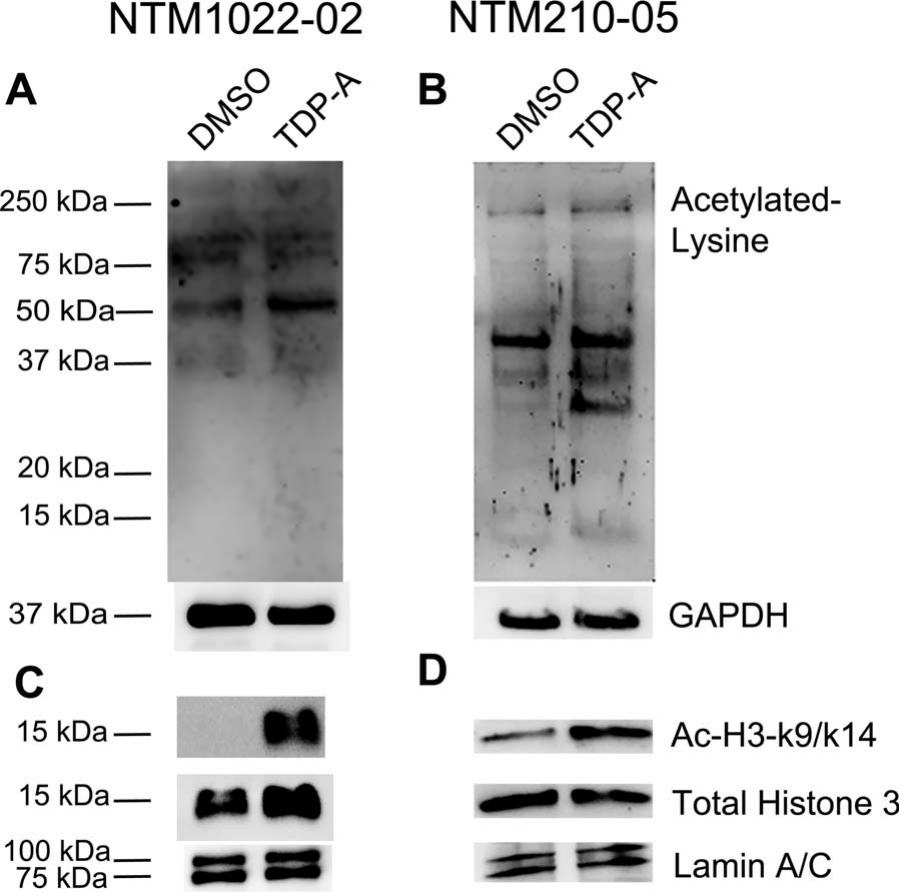
Histone deacetylase inhibitor TDP-A increased protein acetylation in primary NTM cells. Western immunoblots for acetylated proteins in primary TM cells treated for 4 days with DMSO or TDP-A. (**A**, **B**) Whole cell lysate was probed with an antibody against proteins with acetylated lysine residues, and GAPDH was used as a loading control. (**C**, **D**) Nuclear proteins were probed with antibodies against Ac-H3-K9/K14, total histone 3, or Lamin A/C. Lamin A/C was used as a nuclear protein loading control.

We studied whether TDP-A was able to increace TGFβ2 expression by enhancing histone acetylation in the NTM. We tested several concentrations and found 10 nM TDP-A to be the most effective in inducing TGFβ2 expression by using qPCR (data not shown). Therefore, we treated primary NTM cells with 10 nM TDP-A or 1% DMSO as a vehicle control for 4 days. Treatment with TDP-A enhanced total protein acetylation, including histone 3 (Fig. 2), showing that TDP-A is an active HDACi for NTM cells.

### TDP-A Increased Acetylation of Histones Associated With the TGFβ2 Promoter

We performed ChIP assays to determine whether histone proteins associated with the promoter region of the TGFβ2 gene became hyperacetylated after TDP-A treatment. Histone proteins have various patterns of acetylation with differential effects on transcription. Among them, Ac-H3-K9/K14 is mostly associated with the enhancement of gene expression. Therefore, we treated primary NTM cells with 10 nM TDP-A or 1% DMSO and used an anti Ac-H3-K9/K14 antibody for our ChIP assays. In all 3 NTM cell strains studied, TDP-A treatment increased acetylation of histones associated with the TGFβ2 promoter, as shown by the enrichment of TGFβ2 promoter using qPCR (Table 2).

**Table 2:**
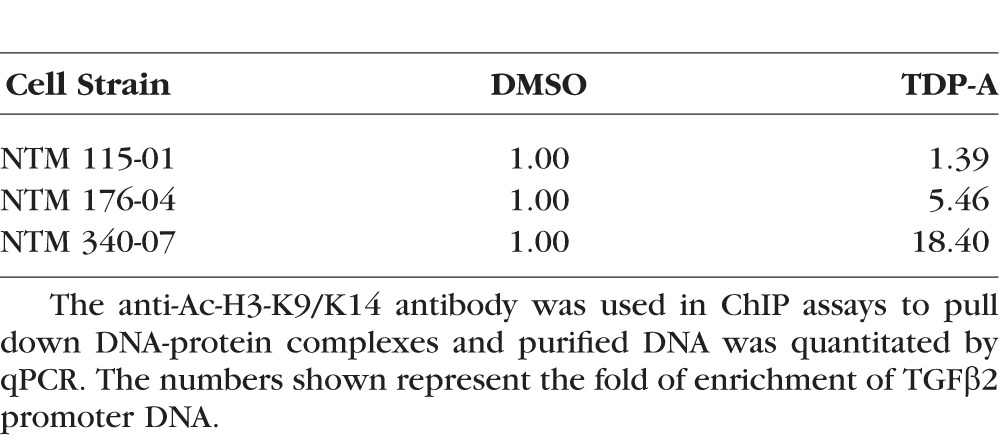
ChIP Assays for Histone Acetylation Associated With the TGFβ2 Promoter

### TDP-A Increased the Expression of TGFβ2 in NTM Cell Cultures

**Figure 3:**
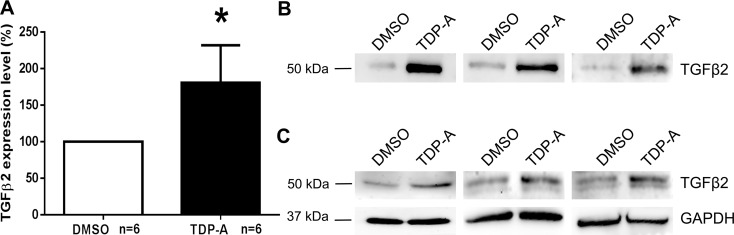
Thailandepsin-A increased the expression of TGFβ2 in cultured primary NTM cells. Nonglaucomatous TM cells were treated with DMSO or TDP-A for 4 days, and the expression of TGFβ2 was compared at mRNA (**A**) and protein (**B**, **C**) levels. (**A**) Quantitative PCR was used to compare the expression of TGFβ2 upon vehicle (DMSO, *open column*) or TDP-A (*shaded column*) treatment. Wilcoxon matched-pairs signed rank test was used for statistical analysis. *P < 0.05, *n* = 6 (NTM 1022–02, 496-05, 340-07, 210-05, 176-04, and 30A). *Columns* and *error bars*: means ± SEM. (**B**) Conditioned medium collected from 3 different NTM cell strains (NTM 1022–02, 210-05, 176-04) was probed for TGFβ2 using WB. (**C**) Whole cell lysates collected from the same NTM cell strains as shown in (**B**) were probed for TGFβ2 using WB.

To determine whether histone hyperacetylation can affect the expression of the glaucoma-associated factor TGFβ2 in the TM, we treated primary NTM cells with 10 nM TDP-A or 1% DMSO as a vehicle control for 4 days. In 6 different NTM cell strains, qPCR analysis showed that TDP-A significantly elevated the expression of TGFβ2 (100.0 ± 0.0% vs. 180.7 ± 51.4%, mean ± SEM; *P* < 0.05; Fig. 3A). In 3 different NTM cell strains, WB analysis showed that TDP-A elevated TGFβ2 in conditioned medium and whole cell lysates (Fig. 3B, 3C, respectively).

### TDP-A Increased IOP, TGFβ2, ECM, and Cytoskeletal Proteins in Perfusion-Cultured Bovine Eyes

Previous studies showed that elevated TGFβ2 in the TM causes ocular hypertension (OHT) in perfusion-cultured human anterior segments and in mouse eyes.^13–16^ Since TDP-A increased the expression of TGFβ2 in TM cells, we studied whether TDP-A is able to elevate IOP as well as alter ECM and cytoskeletal proteins. We used the bovine anterior segment perfusion culture model to determine the effects of TDP-A on IOP. One of the paired bovine eyes was treated with 1% DMSO vehicle and the fellow eye was treated with 10 nM TDP-A for 5 to 9 days.

**Figure 4:**
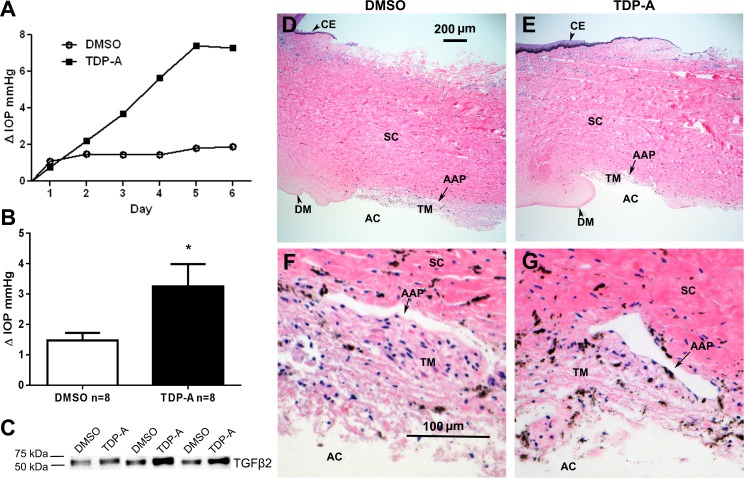
Thailandepsin-A increased IOP and TGFβ2 in perfusion-cultured bovine anterior segments. (**A**) Representative IOPs of a pair of bovine eyes perfused with or without TDP-A. (**B**) Mean changes in IOP after treatment with vehicle (DMSO, *open column*) or TDP-A (*shaded column*). Paired *t*-test was used for statistical analysis. *P < 0.05; *n* = 8. *Columns* and *error bars*: means ± SEM. (**C**) Perfusate (conditioned medium) collected from 3 pairs of bovine eyes was probed for TGFβ2 using WB. After 7-day perfusion culture, bovine anterior segments treated with DMSO (**D**, **F**) or TDP-A (**E**, **G**) were subjected to H&E staining to study potential morphological changes. (**D**, **E**) ×40 magnification. (**F**, **G**) ×200 magnification. AAP, angular aqueous plexus; AC, anterior chamber; CE, cornea epithelium; DM, Descemet's membrane; SC, sclera.

**Figure 5:**
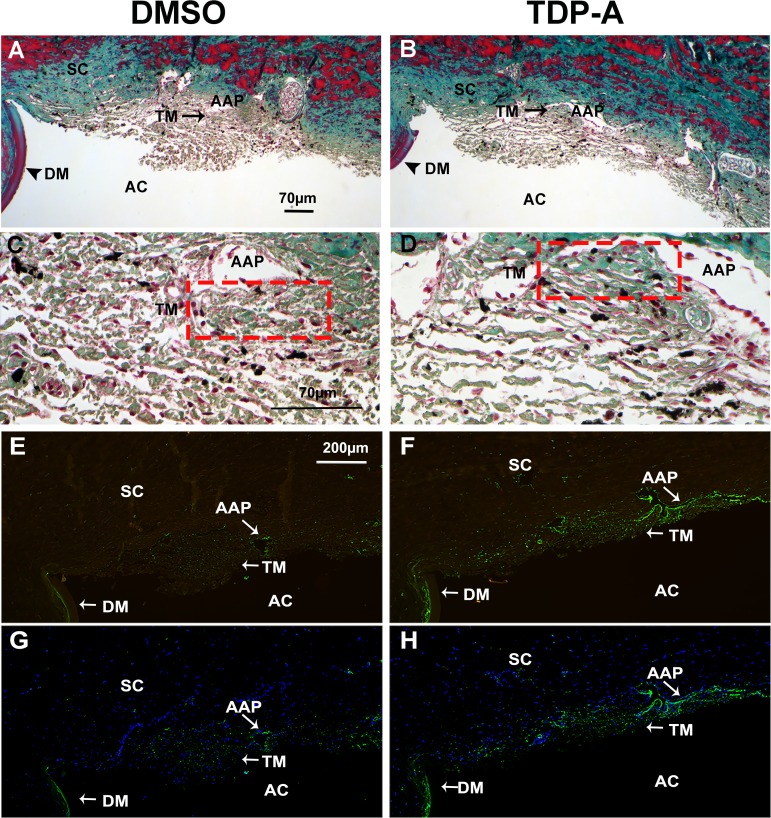
Thailandepsin-A increased collagen and fibronectin expression in perfusion-cultured bovine anterior segments. Perfusion-cultured bovine eyes were treated with vehicle (DMSO) or TDP-A for 7 days. At the end of perfusion, bovine anterior segments were fixed, embedded in paraffin, sectioned and used for Masson's trichrome stain for total collagen (**A**–**D**) as well as immunofluorescent staining for fibronectin (**E**–**H**). (**A**–**D**) Total collagen shown in *blue*-*green*. (**A**, **B**) ×100 magnification. (**C**, **D**) high magnification (×400) of (**A**) and (**B**), respectively. *Red dotted boxes*: TM. (**E**, **F**) images taken using a long pass FITC filter. Fibronectin is shown in *green*, and autofluorescence is shown in *yellow*-*brown*, which helps to identified nonimmunostained tissues. (**G**, **H**) The same images as in (**E**) and (**F**) after autofluorescence subtraction (please see Materials and Methods for details) merged with DAPI staining (*blue*). (**E**–**H**) ×100 magnification.

**Figure 6:**
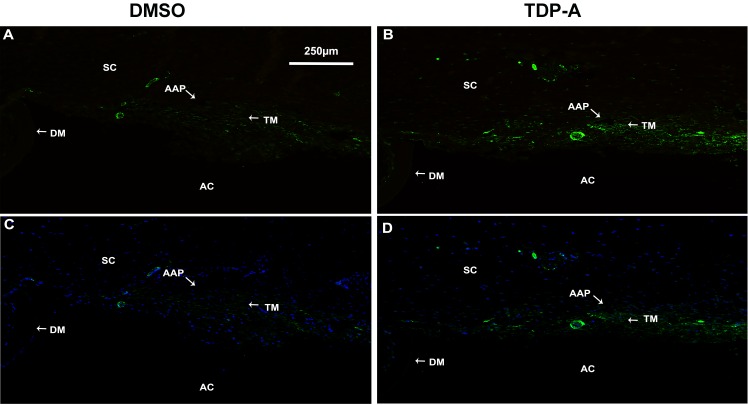
Thailandepsin-A increased α-SMA in perfusion-cultured bovine anterior segments. Perfusion-cultured bovine eyes were treated with vehicle (DMSO) or TDP-A for 7 days. At the end of perfusion, bovine anterior segments were fixed, embedded in paraffin, sectioned, and stained for α- SMA (*green*). Nuclei were stained with DAPI (*blue*). Magnification: ×100. (**A**, **B**) images were taken using a long pass FITC filter. Autofluorescence is shown in *yellow*-*brown*, which helps to identify nonimmunostained tissues. (**C**, **D**) the same images as in (**A**) and (**B**) after autofluorescence subtraction (please see Materials and Methods for details) merged with DAPI staining (*blue*).

We found that TDP-A–treated eyes had significantly higher IOP compared to DMSO–treated eyes (ΔIOP 3.25 ± 0.74 vs. 1.48 ± 0.25 mm Hg, mean ± SEM; *n* = 8, *P* < 0.05; Figs. 4A, 4B). Western immunoblotting of perfusate (conditioned medium) also showed higher TGFβ2 protein expression in TDP-A treated eyes (Fig. 4C). To determine if DMSO or TDP-A caused any obvious morphologic changes to the bovine TM, we collected perfused bovine anterior segments for histologic studies. Light microscopic examination of H&E-stained TM tissue sections showed no apparent changes to the TM in either DMSO or TDP-A treated eyes (Figs. 4D–G). In contrast, Masson's trichrome and immunofluorescent staining showed that TDP-A increased the expression of ECM components, including collagen and fibronectin, in the TM of perfusion-cultured bovine anterior segments (Fig. 5). Immunofluorescent staining also showed an increase in α-SMA expression in the TDP-A–treated bovine TM (Fig. 6).

### The TGFβ Receptor I Inhibitor LY364947 Blocked TDP-A–Induced IOP Elevation in Perfusion-Cultured Bovine Eyes

Although TDP-A elevated IOP in perfusion-cultured bovine eyes, it is possible that this IOP elevation is mediated via other pathways or mechanisms. To rule out this possibility, we cotreated perfusion-cultured bovine eyes with TDP-A and LY364947, a small molecule that inhibits TGFβ signaling via preventing phosphorylation of the TGFβ receptor I. Without TGFβ receptor phosphorylation, the entire TGFβ pathway, including the Smad-dependent and -independent pathways, remain inactive. Our previous studies showed that LY364947 is a potent inhibitor that blocks both TGFβ signaling pathways in the TM.^29,30^

**Figure 7:**
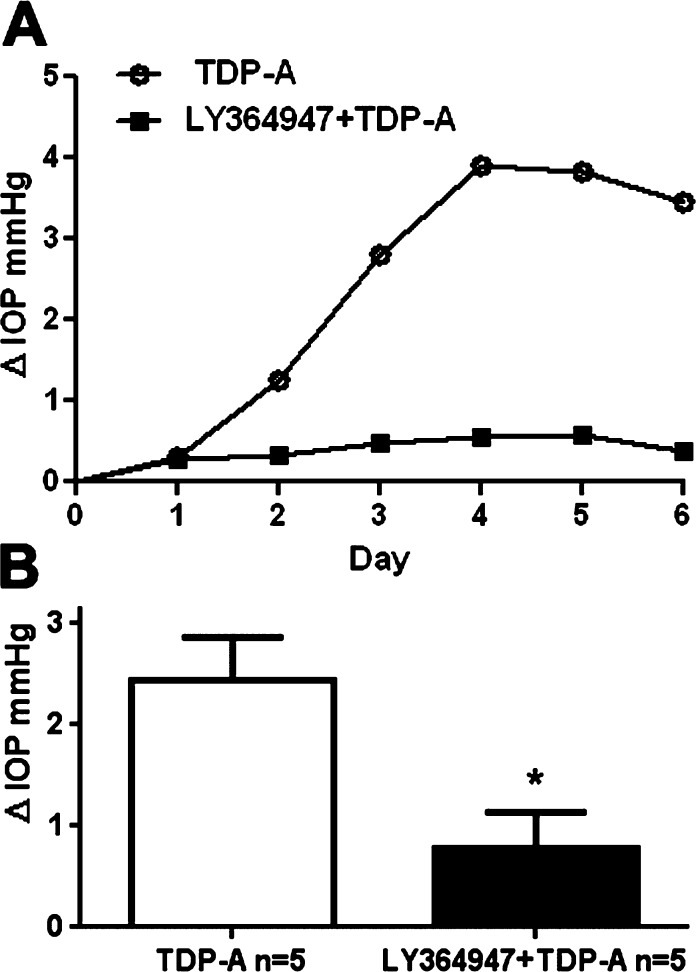
The TGFβ receptor I inhibitor LY364947 prevented TDP-A induced IOP elevation. (**A**) Representative IOPs of a pair of bovine eyes treated with TDP-A ± TGFβ signaling inhibitor LY364947. (**B**) The IOP of perfusion-cultured bovine eyes treated with TDP-A alone (*open column*) or TDP-A + LY364947 (*shaded column*). Paired *t*-test was used for statistical analysis. *P < 0.05; *n* = 5. *Columns* and *error bars*: means ± SEM.

One of the paired bovine eyes was treated with TDP-A as a positive control to induce OHT, while the fellow eye was treated with TDP-A plus LY364947. The experiment was repeated in 5 pairs of bovine eyes. The eyes that were treated with TDP-A plus LY364947 showed significantly lower IOP (ΔIOP 2.45 ± 0.42 vs. 0.78 ± 0.36 mm Hg, mean ± SEM; *n* = 5; *P* < 0.05; Fig. 7), showing that TDP-A–induced IOP elevation is via elevated TGFβ2 expression and associated TGFβ signaling.

## Discussion

In this study, we found that there is hyperacetylation at the lysine residues, especially histone 3 at lysine residues 9 and/or 14 in GTM cells compared to NTM cells. Additionally, we used in vitro and ex vivo models to demonstrate that TDP-A induced histone hyperacetylation in the TM, which increased the expression of glaucoma-associated factor TGFβ2 as well as elevated IOP. Many studies have shown that the expression of TGFβ2 in the aqueous humor of POAG patients is elevated by approximately 50% to 100% compared to control individuals.^31^ The same studies also showed that there is a wide range of TGFβ2 expression in nonglaucomatous individuals, which may explain the variation in histone acetylation seen in our ChIP data (Table 2). Furthermore, the IOP elevation we observed was TGFβ2-dependent because it could be blocked by a TGFβ receptor inhibitor. In the tissue of perfusion-cultured anterior segments, we found that TDP-A increased total collagen, fibronectin, and α-SMA expression. In the TM, these changes have been reported previously to be TGFβ2 inducible,^7,12,32^ further suggesting that our findings were very likely to be caused by TDP-A–induced TGFβ2 elevation in bovine eyes. The HDAC inhibitor TDP-A–induced IOP elevation was 3.25 mm Hg, which was statistically significant in the bovine anterior segment perfusion culture model. In our previous studies using the same model to investigate glucocorticoid-induced glaucoma, we found dexamethasone elevated IOP by more than 2.82 mm Hg.^24^ In this study, TDP-A elevated IOP by more than 3 mm Hg, which is statistically and functionally significant. Taken together, our results showed that histone hyperacetylation may have an important role in the increased expression of glaucoma-associated factor TGFβ2. To our knowledge, this is the first report showing that abnormal histone acetylation may initiate the glaucoma pathologic changes to the TM.

Epigenetic regulation has been shown to have vital roles in a number of diseases. DNA methylation is associated with gene suppression. In cancers, DNA hypermethylation decreases the expression of tumor suppressor genes, which leads to tumorigenesis.^33^ In Alzheimer's disease, changes in global and/or gene-specific DNA methylation have been reported.^34^ However, in POAG, we found that DNA methylation is not the regulatory mechanism responsible for the increased expression of the glaucoma-associated factor sFRP1 in the TM.^21,35^ MicroRNA regulation also contributes to cancers and neurodegenerative diseases. Different from artificial siRNA, miRNA silences a number of genes. Differential expression of different miRNAs, such as miR-15, -16 and miR-21, -155 have been found to suppress or promote tumors, respectively.^36^ In the eye, Gonzalez et al.^37^ showed that miR-200c may have a role in TM cell contraction and IOP regulation.

In contrast to DNA methylation and miRNA, which inhibit gene expression, histone acetylation generally increases gene expression. This modification unwinds chromatin, facilitates transcription factor and cofactor binding, and enhances gene transcription. The balance of histone acetylation is maintained by HDACs and histone acetyl transferases (HATs). In chronic obstructive pulmonary diseases, a profound loss of HDAC2 has been reported, which may mediate the upregulation of inflammatory genes and lower responses to steroids.^38^ In Parkinson's disease, hyperacetylation of H3 or H4 are the key findings in dopaminergic neurons, which may promote neuronal apoptosis.^20^ Nickels et al.^39,40^ showed that HDAC3 and 4 participate in retinal ganglion cell apoptosis in experimental models of glaucoma.

Although HDACs can have positive or negative roles in diseases, most studies showed that HDAC inhibitors are protective, especially in neural diseases. The mechanism by which HDAC inhibitors protect cells or neurons still is not fully understood. They may enhance the expression of molecules, such as neurotrophins and their receptors, Bcl-2, AKT, and nestin that promote neuronal survival, inhibit apoptosis, or induce neural regeneration.^41^ Under experimental glaucomatous conditions, valproic acid (another HDACi) has been shown to protect RGCs in rat ischemia/reperfusion and ocular hypertension models.^42,43^ In DBA/2J mice, a pigmentary glaucoma model that develops ocular hypertension, trichostatin (another HDACi) improves the expression of a subset of RGC genes that are downregulated during this disease process.^44^ Since HDAC inhibitors change global gene expression, beneficial and detrimental genes may be affected. However, it is possible that the promoters of the genes that are involved in disease pathways are hypoacetylated compared to other genes. Therefore, these disease-associated genes are more likely to be rescued by HDACi treatment, while the expression of other disease-irrelevant genes may not change. This may explain why HDAC inhibitors have various therapeutic effects, such as enhancement of cell survival as well as inhibition of tumor growth. In contrast, we found that HDAC inhibitors may adversely upregulate the expression of glaucoma-associated factors such as TGFβ2, leading to elevated IOP. In the TM, HDAC-induced expression of TGFβ2 appeared responsible for the TDP-A–induced OHT. Therefore, the overall effect of HDAC inhibitors is very likely to be tissue- and disease-dependent and should be determined on a case-by-case basis. Also, how TDP-A affects GTM cells may be different from our NTM data, which needs further investigation.

Histone deacetylase inhibitors have five classes and multiple isomers. They not only modify histones, but also bind to and/or modify other transcription factors.^20^ It will be very interesting to find out which HDAC(s) is specific for the regulation of TGFβ2 as well as other glaucoma-associated factors in the TM. Additionally, similar to glaucomatous TM cells, glaucomatous lamina cribrosa (LC) cells, a specialized cell type in the optic nerve head (ONH) that is a primary site of glaucomatous optic neuropathy, have been shown to have increased expression of TGFβ2.^26,45^ Glaucomatous LC cells also demonstrate increased ECM deposition and cytoskeletal changes that can be induced by TGFβ2 just like TM cells.^26,46,47^ Thus, it is possible that histone hyperacetylation also is responsible for the increased expression of TGFβ2 in the glaucomatous ONH.

Since we found that an HDACi elevated IOP, it is reasonable to speculate that histone acetyl transferase inhibitors would decrease TGFβ2 expression and lower IOP. Histone acetyl transferases are enzymes that increase histone acetylation. Similar to HDACs, they also can bind to other transcription factors and regulate gene expression. Histone acetyl transferases generally function as transcriptional coactivators. The 17 human HATs are divided into five classes.^48^ In contrast to the diversity of various HDAC inhibitors, not many HAT inhibitors are available. Several HAT inhibitors, such as anacardic acid and curcumin, have been shown to inhibit Jurkat T-cell leukemia cells and prevent heart failure in rats, respectively.^49,50^ Kanthasamy et al.^51^ showed that anacardic acid attenuated dieldrin-induced damage in primary mesencephalic cells.^51^ Like HDAC inhibitors, HAT inhibitors may affect genes that are selectively activated in POAG. Thus, HAT inhibitors may be a potential therapeutic approach in treating POAG.

We have provided evidence supporting the role of epigenetic regulation of TGFβ2 as a glaucoma mediator that elevates IOP. However, other glaucoma-associated factors such as sFRP1,^52^ gremlin,^7^ serum amyloid A,^52^ sCD44,^53^ and cochlin,^54^ also may be epigenetically regulated to elevate IOP. Although these factors have been elevated in POAG AH and/or TM, to our knowledge, there have not been any studies to find out whether some or all of them are coexpressed in POAG patients. If that is true, it is very likely that the other glaucoma-associated factors are elevated in POAG via a similar epigenetic mechanism as TGFβ2.

**Figure 8:**
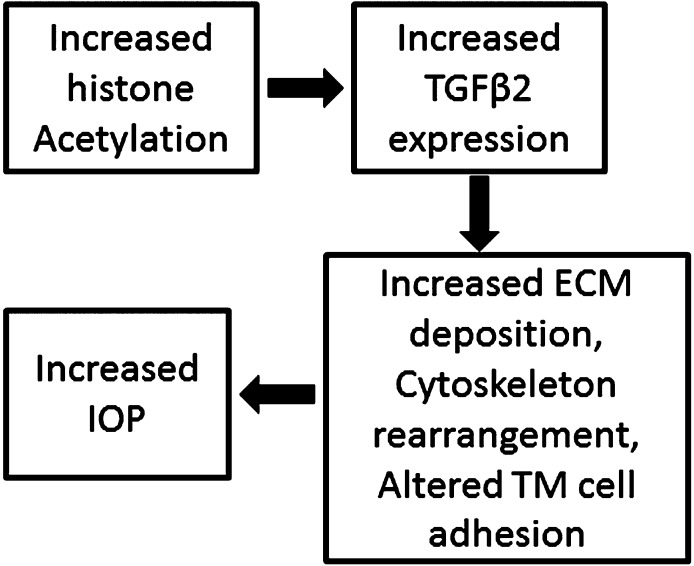
Schematic of histone acetylation in IOP regulation.

In summary, our findings suggested epigenetic regulation as a novel mechanism in TM pathogenesis and IOP regulation (Fig. 8). Further research is needed to help better understand the role of epigenetics in POAG. Our discovery may provide novel therapeutic strategies and targets for treating glaucoma.
